# Necrology

**Published:** 1903-02

**Authors:** 


### NECROLOGY.

JOHN AIRTH, M.R.C.V.S., of Sioux City, Iowa, died in November last from asphyxia, due to his inadvertently turning on the gas in a stove which he used in his room for heating purposes. His funeral was conducted by the Sioux City Thistle Club, of which he was a member, and it was largely attended by his numerous friends and acquaintances. Dr. Airth was one of the pioneer members in the Government Service, holding the position of Meat Inspector for two years. During the past year he made a trip to South Africa in charge of a shipload of mules. On his return trip he visited his old home at Dumfriesshire, Scotland. Shortly after his return he met with this fatal accident. For the past seven years he was in active practice at Sioux City, Iowa, and was a very successful and popular veterinarian.

